# Imprint Desorption Electrospray Ionization Mass Spectrometry Imaging (IDESI-MSI) Reveals Absorption of Triclopyr-Based Herbicide in Plants and Mouse Organs

**DOI:** 10.3390/metabo15070437

**Published:** 2025-06-30

**Authors:** Hanzhi Liu, Yunshuo Tian, Ruolun Wei, Yifan Meng, Richard N. Zare

**Affiliations:** 1 College of Letters and Science, University of California, Santa Barbara, CA 93106, USA; 2 Department of Neurosurgery, School of Medicine, Stanford University, Stanford, CA 94305, USA; rlwei@stanford.edu; 3 Department of Chemistry, Stanford University, Stanford, CA 94305, USA

**Keywords:** imprint desorption electrospray ionization mass spectrometry imaging, oil-absorbing film, herbicide accumulation

## Abstract

Background: Understanding the absorption and distribution of herbicides in plants and animal tissues is essential for assessing their potential risks to human health. Method: In this study, we employed imprint desorption electrospray ionization mass spectrometry imaging (IDESI-MSI) to visualize in both vegetable and animal tissues the absorption of Roundup which is a widely used herbicide. Results: Using IDESI-MSI with a pixel size of 150 µm, we detected the herbicide alongside several endogenous metabolites on oil-absorbing films applied to carrot sections. Time-course experiments revealed progressive herbicide penetration into carrot tissue, with penetration depth increasing linearly over time at a rate of approximately 0.25 mm/h. In contrast, green pepper samples showed minimal herbicide infiltration, likely owing to their hydrophobic cuticle barrier. Additionally, mice fed with herbicide-treated carrots exhibited detectable levels of herbicide in liver and kidney tissues. Conclusions: These findings highlight the utility of IDESI-MSI as a powerful analytical platform for the rapid evaluation of chemical migration and absorption in food and biological systems, with important implications for food safety and toxicological research.

## 1. Introduction

The widespread use of herbicides in modern agriculture has raised concerns about their potential to penetrate edible plant tissues and accumulate in body organs after ingestion by animals and humans [1,2,3]. Roundup, which is a popular herbicide, is widely used for its broad-spectrum weed control [4,5,6]. At one time Roundup was used to refer to glyphosate-based herbicides, but now there are other formulations of herbicides that are also marketed as Roundup [7]. For example, Roundup Dual Action is a formulation that includes triclopyr, [(3,5,6-trichloro-2-pyridinyl)oxy] acetic acid, as the active ingredient. The widespread use of the herbicide in agriculture has raised increasing concerns about its residue stability in food and its potential health effects [8,9]. Conventional analytical techniques, such as liquid chromatography–mass spectrometry (LC-MS) [10,11,12,13], can quantify the total amount of herbicide residues in plant or animal samples, but cannot provide spatially resolved information. As a result, these methods cannot reveal the depth of herbicide penetration or precise localization in tissues. Analytical methods with spatial resolution are indispensable for understanding chemical transport mechanisms and for assessing potential exposure risks.

Mass spectrometry imaging (MSI) is a powerful surface chemical imaging technique for visualizing the spatial distribution of small molecules, metabolites, and exogenous compounds in complex biological systems [14,15,16]. Among various MSI techniques, desorption electrospray ionization mass spectrometry imaging (DESI-MSI) has the advantages of atmospheric pressure operation and easy sample pretreatment, which makes it particularly suitable for research in biology and agricultural science [17,18,19]. The imprint-based DESI-MSI (IDESI-MSI) method further simplifies the experimental process by transferring analytes from the sample surface to a flexible film for subsequent analysis [20,21]. This method has been successfully applied to the imaging of endogenous metabolites in tissues and plants, as well as to the visualization of environmental contaminants on surfaces [22,23,24,25]. In addition to DESI-MSI, the imprint method can also be used in laser-based MSI techniques, which has been well shown in several studies [24,26].

In our previous work, oil-absorbing film has been proven to be an efficient imprint material, owing to its low cost and high transfer efficiency of both small metabolite molecules as well as lipids [27]. In this study, we applied oil-absorbing-film-based IDESI-MSI to investigate the surface penetration behavior of Roundup herbicide and its incorporation process in plant and animal tissues. Environmental exposure scenarios were simulated by spraying a Roundup solution onto the surface of carrots, and the spatial distribution of the herbicide and endogenous metabolites was visualized using MSI. Temporal gradient experiments showed that the penetration of herbicides in tissues progressively deepened with exposure time, and the rate of penetration could be calculated accordingly. In contrast, the green pepper samples showed almost no significant penetration, probably caused by the effective barrier formed by their hydrophobic external cuticle and epicuticular waxes [28]. In addition, significant herbicide signals were detected in both liver and kidney tissues from mice fed with Roundup-treated carrots. The above results indicate that the IDESI-MSI technique can be used to evaluate transfer behavior, permeation barrier effect, and systemic incorporation of chemical substances, providing a powerful analytical tool for food safety monitoring and environmental toxicology studies.

## 2. Materials and Methods

### 2.1. Chemicals and Reagents

Round herbicide spray (Bayer, Leverkusen, Germany) was purchased from a local agricultural supply store. The concentration of the active molecule (triclopyr) is 0.122% according to the label (Figure S1). Acetonitrile (ACN) was purchased from Sigma-Aldrich (St. Louis, MO, USA) and mixed with water in an 8:2 volume ratio for use as the DESI solvent.

### 2.2. Plant Sample Preparation and Treatment

Fresh carrots and green peppers were purchased from a local grocery store (Palo Alto, CA, USA). Carrots were exposed to the diluted Roundup solution without any pre-treatment at room temperature. After incubation, the carrots were sliced longitudinally to expose the internal cross-section. Oil-absorbing film (Clean & Clear^®^, Johnson & Johnson, New Brunswick, NJ, USA) was pressed against the cross-section for 10 s to obtain an imprint of the carrot section. For green pepper experiments, the outer surface was similarly sprayed with the Roundup solution and allowed to incubate for 0.5 to 12 h. After incubation, the pepper was cut open, and the cross-section was imprinted as described above.

### 2.3. Animal Feeding and Tissue Collection

To investigate systemic incorporation, a female C57BL/6 mouse (6 weeks old) was kept under standard laboratory conditions and fed slices of 12 h-Roundup-treated carrot for 48 h. By weighing the carrots before and after feeding, the total weight of carrots consumed by the mice within 48 h was approximately 5.2 g. After treatment, the mouse was euthanized, and liver and kidney tissues were harvested. Liver and kidney were frozen-sectioned with a knife to expose fresh surfaces, and oil-absorbing film was applied with uniform pressure for 10 s to obtain imprints for MSI analysis. The control group was fed with fresh carrots (no herbicide-exposed), while other conditions were the same to the treated group.

### 2.4. Imprint Desorption Electrospray Ionization Mass Spectrometry Imaging (IDESI-MSI)

DESI-MSI experiments were performed using a commercial DESI sprayer (Viktor Tech, Beijing, China) coupled to an Orbitrap Velos Pro mass spectrometer (Thermo Fisher Scientific, Waltham, MA, USA). The DESI spray capillary had an inner diameter of 20 µm and an outer diameter of 120 µm. The sprayer was positioned at a 60° angle relative to the sample surface, with the capillary-to-surface distance set to 4 mm and the capillary-to-inlet distance at 2 mm. Compressed nitrogen (99.999%) was used as the nebulizing gas at 120 psi. A high-voltage of −6 kV was applied to the sprayer, and the solvent flow rate was maintained at 1.5 µL/min. Full-scan MS data were acquired in negative ion mode over an *m/z* range of 100–1000, with a resolution of 30,000. The scan rate was fixed by disabling automatic gain control (AGC). The maximum injection time was set at 150 µs, and the ion transfer capillary temperature was 300 °C. Imaging was performed by raster-scanning the sample stage at a speed of 600 µm/s along the *X*-axis with a step size of 150 µm along the *Y*-axis.

### 2.5. Data Analysis

Mass spectrometry data were acquired using Xcalibur 1.4 (Thermo Fisher Scientific) software, with each row of the image saved as an independent .raw file. These .raw files were converted to .cdf files using Xcalibur and then imported into a custom-written MATLAB R2024b program for visualization. All images were normalized to the percentage of total ion current (TIC). The *m/z* tolerance for the images was set to ±5 ppm to match the expected mass accuracy of the Orbitrap. This method enables semi-quantitative analysis of the TIC-normalized intensities of various small-molecule metabolites, lipids, and herbicides within plant sections. The penetration depth of herbicides is estimated to be based on the spatial distribution coordinates of the herbicide mass spectrometry signals (150 μm/pixel). The maximum penetration was determined by setting a threshold (20%) signal intensity of herbicide and measuring the point at which this signal no longer increases. During the penetration of herbicides into plants, uneven penetration may occur on the same plane. Therefore, the penetration depth of herbicides is simulated by calculating the average depth of multiple penetration paths (Figure S2).

## 3. Results

### 3.1. IDESI-MSI of Carrot Exposed to Herbicide

**Figure 1:**
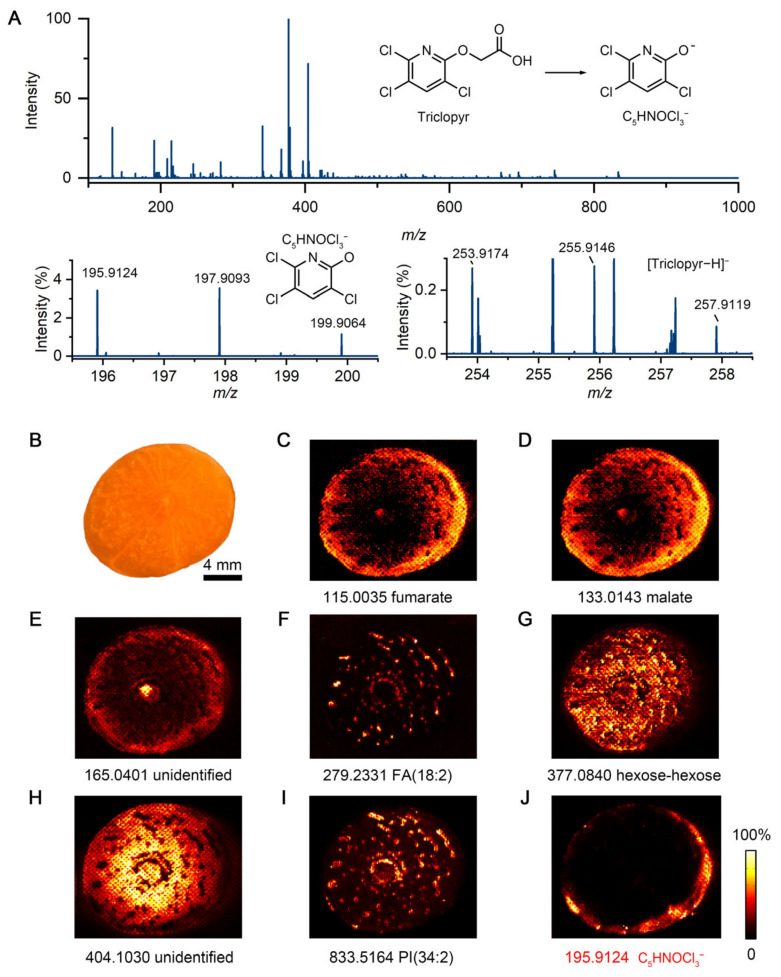
Imprint desorption electrospray ionization mass spectrometry imaging (IDESI-MSI) of carrot exposed to Roundup herbicide for 2 h. (**A**) A typical mass spectrum of the imprint of carrot section. Peaks of herbicide (fragments and mother ion) are shown in the zoomed-in spectrum. (**B**) Optical image of the carrot section that was imprinted to the oil-absorbing film. (**C**–**I**) IDESI-MSI images of metabolites and lipids from the imprint. (**J**) IDESI-MSI image of the peak at *m/z* 195.9124 from the herbicide.

To investigate the penetration behavior of herbicides in plant tissue, we applied IDESI-MSI to visualize the molecular distribution on the surface and interior of carrot exposed to herbicide. A carrot was sprayed with herbicide solution and incubated for 2 h under room temperature. The carrot was then sliced longitudinally about 3 cm from the root, and the internal surface was pressed against an oil-absorbing film to obtain an imprint for DESI-MSI analysis (Figure S3). Figure 1 shows IDESI-MSI images obtained from the imprint of the carrot cross-section. Several endogenous molecules from the carrot were detected by MS, such as metabolites, organic acids, and lipids (Figure 1A). According to the user information of the Roundup herbicide, one of the active ingredients is triclopyr (structure shown in Figure 1A, herbicide label shown in Figure S1). As shown in the zoomed-in mass spectra, the deprotonated peaks of triclopyr (C_7_H_3_Cl_3_NO_3_^−^) at *m/z* 253.9174, 255.9146, and 257.9119 can be observed from the scanning of the imprint of carrot section. However, the fragment peaks (C_5_HNOCl_3_^−^) at *m/z* 195.9124, 197.9093, and 199.9064 showed higher intensity. This might be due to the in-source fragmentation effect because of the high voltage applied to the DESI sprayer (−5 kV) or the high temperature applied to the ion transfer capillary (300 °C). It has been reported that C_5_HNOCl_3_^−^ is a stable fragment of triclopyr [29]. Also, the nanoESI spectrum of pure Roundup herbicide confirms the same assignment of C_5_HNOCl_3_^−^ (Figure S4). Taken together with these findings, the fragment peak of C_5_HNOCl_3_^−^ at *m/z* 195.9124 was used to identify the distribution of Roundup herbicide in the following MSI experiments.

Figure 1B shows the optical image of the carrot cross-section, which was imprinted to the oil-absorbing film. A perceptually linear color was used as the color scale to show the MS images [30]. As shown in Figure 1C–I, several ion images of different molecules from the carrot section can be obtained from the imprint by IDESI-MSI. For example, fumarate at *m/z* 115.0035 is mainly detected in the cortex of the carrot (Figure 1C). Similarly, malate at *m/z* 133.0143 is also distributed in the cortex area as shown in Figure 1D. An undefined metabolite at *m/z* 165.0401 mainly located in the core and cortex, which shows different intensity distribution to malate and fumarate (Figure 1E). Notably, FA (18:2) at *m/z* 279.2331 and PI (38:5) at *m/z* 833.5164 are distributed in a star-like pattern, so it is likely that they are primarily located in the vascular bundles of the carrot (Figure 1F,I). As demonstrated in Figure 1G,H, hexose-hexose at *m/z* 377.0840 and another undefined molecule at *m/z* 404.1030 show a complementary positional distribution to FA (18:2) and PI (38:5). These molecules detected in this study, which also have been studied in previous research [31,32], were mainly identified based on *m/z* matching. The *m/z* ppm errors of peaks shown in this study are provided in Table S1.

As shown in Figure 1J. it can be clearly observed that herbicide had penetrated from the surface into the interior of the carrot at 2 h treatment time. The localization of herbicide signals and endogenous metabolites suggests that the imprint approach efficiently captures both exogenous molecules and native molecular profiles from the same tissue interface. These results illustrate that IDESI-MSI can be used to detect herbicide penetration into plant tissues and to distinguish between endogenous metabolites and externally introduced compounds.

### 3.2. Time-Dependent Accumulation of Herbicide in Carrot

**Figure 2:**
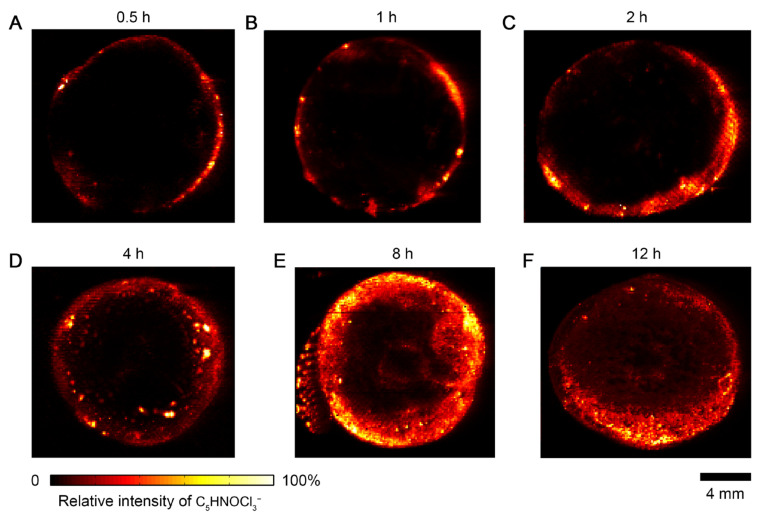
Imprint desorption electrospray ionization mass spectrometry imaging (IDESI-MSI) of herbicide in carrot exposed to Roundup for different time. (**A**–**F**) Exposure time from 0.5 h to 12 h.

To evaluate the kinetics of herbicide penetration in plant tissue, six carrots were exposed to Roundup herbicide and incubated for 0.5, 1, 2, 4, 8, and 12 h, respectively. After each incubation period, the carrot was sliced and imprinted onto oil-absorbing film for DESI-MSI analysis. Figure 2 presents images of the herbicide acquired from these imprints. Each image was individually scaled while no thresholding was applied. At the 0.5 h time point, the herbicide signal was largely confined to the surface layer of the carrot, indicating limited penetration (Figure 2A). By 1 h, the signal had expanded into the inner cortex and a moderately strong distribution was observed close to the central vascular region (Figure 2B). After 2 h of treatment, the herbicide signal had been widely distributed in the peripheral and internal regions of the carrot tissue (Figure 2C). The above spatial changes clearly indicate that the process of herbicide diffusion from the surface to the interior is significantly time dependent. As shown in Figure 2D–F, the herbicide spreads to the center region of the carrot slices as the exposure time increases. The distribution of malate, FA(18:2), hexose-hexose, and PI(34:2) in carrot exposed to the herbicide for 8 h and 12 h are shown in Figure S5, which indicates that the penetration of herbicide does not influence the distribution of these metabolites.

**Figure 3:**
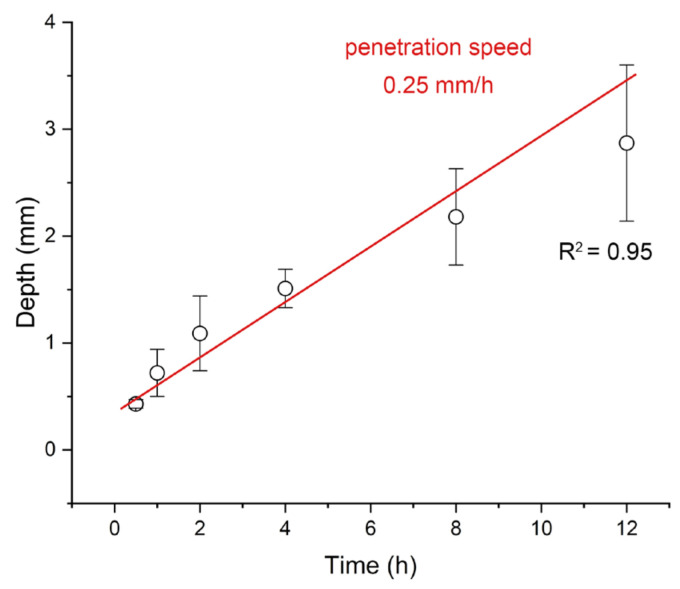
Calculation of the penetration speed of Roundup herbicide in carrot from outer peel to inner core.

To quantitatively estimate the penetration depth and rate of the herbicide, the distribution profile of herbicide signal intensity was extracted along the cross-sectional axis for each time point. We extracted the intensity profile of herbicide ions along the profile axis (see Section 2.5). The penetration depth of herbicides is estimated to be based on the spatial distribution coordinates of the herbicide mass spectrometry signals (150 μm/pixel). During the penetration of herbicides into plants, uneven penetration may occur on the same plane. Therefore, the penetration depth of herbicides is simulated by calculating the average depth of multiple penetration paths. After converting pixels to depth, as shown in Figure S1, it can be seen that penetration depth increased linearly with exposure time. As shown in Figure 3, the average penetration depth increased with incubation time and followed an approximately linear trend. A linear fit result yielded a penetration rate of ~0.25 mm/h.

In this study, we used fresh-harvested carrot and bell pepper tissues rather than whole plants grown in soil. Although isolated tissues lack biological processes such as transpiration and metabolism found in living plants, their natural cuticle, cell wall structure, and intercellular spaces remain intact. These structures determine the initial passive diffusion process of pesticides into plant tissues. Passive diffusion through the cuticle and cortex has been widely recognized as the first step in herbicide absorption [33]. In living plants, subsequent processes such as vascular transport and enzymatic metabolism further transform the herbicide; however, our experimental protocol focused on the physical-chemical diffusion component, which can be compared to the absorption of herbicides by plants after spraying. The penetration rate we measured (~0.25 mm h^−1^) under ambient conditions is in the same range that has been reported before [34]. In agricultural practice, environmental factors (temperature, humidity) and transpiration from living plants can accelerate or slow down the absorption of herbicides. The rate we measured can be regarded as a simulation of the level of herbicide absorption by plants after herbicide application in the field.

### 3.3. IDESI-MSI of Green Pepper Exposed to Herbicide

**Figure 4:**
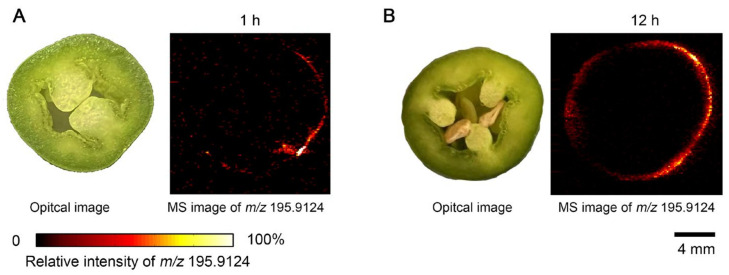
Imprint desorption electrospray ionization mass spectrometry imaging (IDESI-MSI) of herbicide in green pepper exposed to Roundup for different time. (**A**) 1 h. (**B**) 12 h.

To verify whether herbicide penetration is influenced by plant surface properties, we performed the same IDESI-MSI analysis on green pepper samples. The outer surface of the peppers was uniformly sprayed with Roundup herbicide and incubated for different times under the same conditions as the carrot experiment. The green pepper was then cut, and the cross-section was imprinted on an oil-absorbing film for subsequent MSI analysis. As shown in Figure 4, unlike in carrot samples, the herbicide ion signal was undetectable in the interior regions of the green pepper, even after 12 h exposure. Only trace signal was observed near the outer epidermal layer, and no significant herbicide intensity was detected beyond the surface.

This result suggests that the waxy surface and hydrophobic epidermis of green peppers are effective in blocking the penetration of aqueous herbicides. The comparison between carrots with good permeability and green peppers with permeation-blocking ability highlights the key role of plant surface properties in determining the permeation behavior of agrochemicals. Therefore, IDESI-MSI provides a convenient platform for the comparison of permeability of different plant tissues and the identification of structural barriers to exogenous compound migration.

### 3.4. Accumulation of Herbicide in Mouse Organs

To assess whether accumulation of herbicide residues in treated food occurs in animal organs, we fed a mouse with herbicide-treated carrot slices and subsequently analyzed its liver and kidney tissues using IDESI-MSI. After 48 h of continuous feeding, the mouse was euthanized and dissected. Freshly cut liver and kidney surfaces were taken for imprinting, and samples were collected using an oil-absorbent membrane and subsequently analyzed by IDESI-MSI.

**Figure 5:**
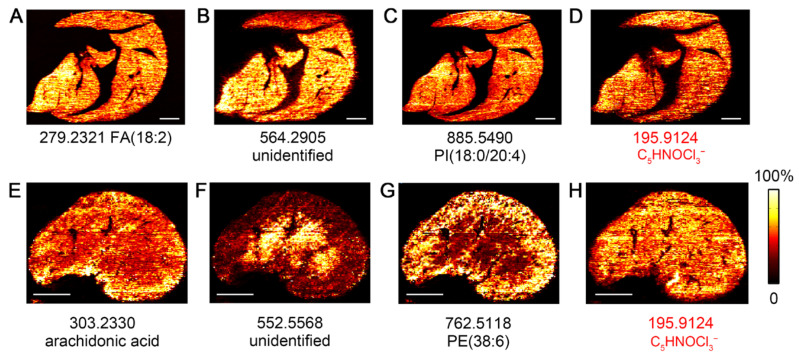
Imprint desorption electrospray ionization mass spectrometry imaging (IDESI-MSI) reveals accumulation of herbicide in mouse organs. (**A**–**C**) IDESI-MSI images of peaks at *m/z* 279.2321, 564.2905, and 885.5490 in mouse liver section. (**D**) MSI image of peak at *m/z* 195.9124 from herbicide in mouse liver section. (**E**–**G**) IDESI-MSI images of peaks at *m/z* 303.2330, 552.5568, and 762.5118 in mouse kidney section. (**H**) MSI image of peak at *m/z* 195.9124 from herbicide in mouse kidney section. Scale bar represents 2 mm in all figures.

**Figure 6:**
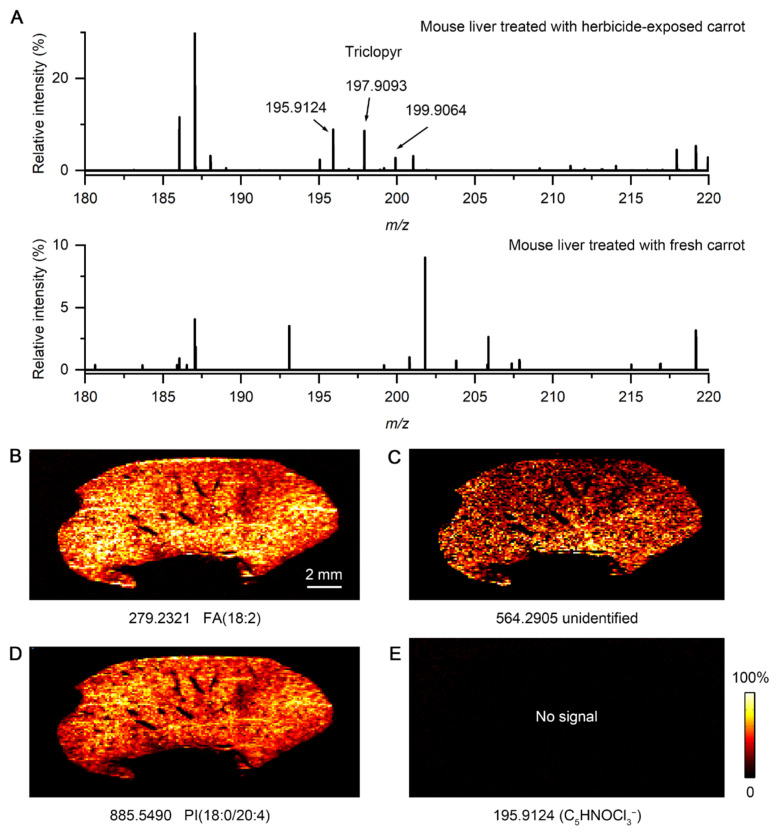
Imprint desorption electrospray ionization mass spectrometry imaging (IDESI-MSI) results of fresh-carrot-treated mouse liver. (**A**) Compared mass spectrum of mouse liver imprint from mouse that was fed herbicide-exposed carrot and fresh carrot. (**B**–**D**) MSI images of peaks at *m/z* 279.2321, 564.2905, and 885.5490. (**E**) MSI image at *m/z* 195.9124 (no signal detected).

As shown in Figure 5, endogenous metabolites and lipids were successfully detected in both liver and kidney imprinted samples, indicating good molecular transfer efficiency and reliable imaging quality. More importantly, herbicide was detected in both organs, with a higher relative intensity in the kidney than in the liver. These signals were diffusely distributed, suggesting that herbicides may be systemically absorbed and distributed in the body through blood circulation. To further validate the assignment of the peak of C_5_HNOCl_3_^−^, we performed a negative-control experiment using a liver imprint from a mouse that had been fed fresh carrots (no herbicide-exposed). As shown in Figure 6A, the triclopyr ion and its chloride isotopes (*m/z* 195.9124, 197.9093, and 199.9064) are clearly observed in the mass spectrum of kidney tissue from a treated mouse, with the expected isotopic ratios. These peaks are not detected in the spectrum of the fresh-carrot-treated mouse liver tissue imprint. Figure 6B–D present IDESI-MSI images of the same ions in the liver imprint corresponding to those shown in Figure 5A–C, while no signal at *m*/*z* 195.9124 was detected in the control (Figure 6E). These results further confirm that the peak at *m/z* 195.9124 that we used to generate the herbicide image in mouse tissues is correctly assigned.

These findings suggest that Roundups accumulate in metabolically active organs once they enter the body through food intake. A careful evaluation of how long the food is in contact with herbicide and how the food is treated is critical in preventing harmful chemicals from entering the human body by ingestion. IDESI-MSI enables the localization and visualization of these exogenous compounds directly on the surface of organ imprints, demonstrating its potential application in toxicological and pharmacokinetic studies. In addition, the method does not require labeling and provides an efficient means to track the absorption of small molecules in biological systems.

## 4. Discussion

In this study, we demonstrated that IDESI-MSI is a powerful tool for visualizing the process of herbicide penetration in plants and absorption in animal tissues. Using carrots as a plant model, we observed a clear time-dependent process of herbicide penetration in plants, allowing an estimation of its molecular penetration rate. In contrast, green peppers showed significant resistance to herbicide uptake, which could be attributed to their hydrophobic cuticle, further indicates structural differences in permeability to pesticides across plants. In addition, we found that herbicide signals can be detected in both liver and kidney tissues of mice that are ingested by herbicide-treated carrots. This result demonstrates the transfer of harmful chemicals from food to animals and their absorption.

## 5. Conclusions

This finding suggests that IDESI-MSI can be used as a non-targeted, label-free method for tracing the migration pathways of environmental contaminants from food to animal organs. Compared to traditional analysis methods, IDESI-MSI can visualize exogenous compounds and endogenous metabolites simultaneously, providing spatial and metabolic contextual information at the molecular level. This method provides a flexible and efficient platform for evaluating the transport process of exogenous chemicals, the barrier function of plant tissues and their systemic accumulation. It also has broad application prospects in the fields of food safety monitoring, pesticide regulation and environmental toxicology.

## Data Availability

Data is available on reasonable requests from corresponding authors.

## Supplementary Materials

The following supporting information can be downloaded at https://www.mdpi.com/article/10.3390/metabo15070437/s1, Figure S1. Label of the herbicide that we used in this study. Figure S2. Method for calculating the average penetration depth. Figure S3. Scheme of the sample preparation and DESI-MSI process. Figure S4. NanoESI mass spectrum of Roundup herbicide in negative ion mode. Figure S5: The distribution of malate, FA(18:2), hexose-hexose, and PI(38:5) in carrot exposed to the herbicide for 8 h and 12 h. Table S1: Theoretical and measured *m/z* of ions detected in this study.

## References

[1] Kniss A.R. (2017). Long-term trends in the intensity and relative toxicity of herbicide use. Nat. Commun..

[2] Singh S., Kumar V., Gill J.P., Datta S., Singh S., Dhaka V., Kapoor D., Wani A.B., Dhanjal D.S., Kumar M. (2020). Herbicide Glyphosate: Toxicity and Microbial Degradation. Int. J. Environ. Res. Public Health.

[3] Vonk J.A., Kraak M.H.S., de Voogt P. (2020). Herbicide Exposure and Toxicity to Aquatic Primary Producers. Reviews of Environmental Contamination and Toxicology Volume 250.

[4] Giesy J.P., Dobson S., Solomon K.R., Ware G.W. (2000). Ecotoxicological Risk Assessment for Roundup^®^ Herbicide. Reviews of Environmental Contamination and Toxicology: Continuation of Residue Reviews.

[5] Williams G.M., Kroes R., Munro I.C. (2000). Safety Evaluation and Risk Assessment of the Herbicide Roundup and Its Active Ingredient, Glyphosate, for Humans. Regul. Toxicol. Pharmacol..

[6] Séralini G.-E., Clair E., Mesnage R., Gress S., Defarge N., Malatesta M., Hennequin D., de Vendômois J.S. (2014). Republished study: Long-term toxicity of a Roundup herbicide and a Roundup-tolerantgenetically modified maize. Environ. Sci. Eur..

[7] When Roundup Isn’t Roundup: Clearing up the Confusion Between Products. https://purduelandscapereport.org/article/when-roundup-isnt-roundup-clearing-up-the-confusion-between-products/.

[8] Owagboriaye F.O., Dedeke G.A., Ademolu K.O., Olujimi O.O., Ashidi J.S., Adeyinka A.A. (2017). Reproductive toxicity of Roundup herbicide exposure in male albino rat. Exp. Toxicol. Pathol..

[9] Bartels M., Brown C., Chung G., Chan M., Terry C., Gehen S., Corvaro M. (2020). Review of the pharmacokinetics and metabolism of triclopyr herbicide in mammals: Impact on safety assessments. Regul. Toxicol. Pharmacol..

[10] Shin E.-H., Choi J.-H., Abd El-Aty A.M., Khay S., Kim S.-J., Im M.H., Kwon C.-H., Shim J.-H. (2011). Simultaneous determination of three acidic herbicide residues in food crops using HPLC and confirmation via LC-MS/MS. Biomed. Chromatogr..

[11] Kaczyński P. (2017). Clean-up and matrix effect in LC-MS/MS analysis of food of plant origin for high polar herbicides. Food Chem..

[12] Pareja L., Cesio V., Heinzen H., Fernández-Alba A.R. (2011). Evaluation of various QuEChERS based methods for the analysis of herbicides and other commonly used pesticides in polished rice by LC–MS/MS. Talanta.

[13] Sack C., Vonderbrink J., Smoker M., Smith R.E. (2015). Determination of Acid Herbicides Using Modified QuEChERS with Fast Switching ESI^+^/ESI^–^ LC-MS/MS. J. Agric. Food Chem..

[14] Xiao X., Guan X., Xu Z., Lu Q. (2024). In-Situ Metabolic Profiling of Different Kinds of *Rheum palmatum* L. by Laser Desorption–Dielectric Barrier Discharge Ionization Mass Spectrometry Imaging. Metabolites.

[15] Wu C., Dill A.L., Eberlin L.S., Cooks R.G., Ifa D.R. (2013). Mass spectrometry imaging under ambient conditions. Mass Spectrom. Rev..

[16] Chughtai K., Heeren R.M.A. (2010). Mass Spectrometric Imaging for Biomedical Tissue Analysis. Chem. Rev..

[17] Takáts Z., Wiseman J.M., Gologan B., Cooks R.G. (2004). Mass Spectrometry Sampling Under Ambient Conditions with Desorption Electrospray Ionization. Science.

[18] Morato N.M., Cooks R.G. (2023). Desorption Electrospray Ionization Mass Spectrometry: 20 Years. Acc. Chem. Res..

[19] Li B., Hansen S.H., Janfelt C. (2013). Direct imaging of plant metabolites in leaves and petals by desorption electrospray ionization mass spectrometry. Int. J. Mass Spectrom..

[20] Tata A., Perez C.J., Ore M.O., Lostun D., Passas A., Morin S., Ifa D.R. (2015). Evaluation of imprint DESI-MS substrates for the analysis of fungal metabolites. RSC Adv..

[21] Wu L., Qi K., Liu C., Hu Y., Xu M., Pan Y. (2022). Enhanced Coverage and Sensitivity of Imprint DESI Mass Spectrometry Imaging for Plant Leaf Metabolites by Post-photoionization. Anal. Chem..

[22] Hemalatha R.G., Ganayee M.A., Pradeep T. (2016). Electrospun Nanofiber Mats as “Smart Surfaces” for Desorption Electrospray Ionization Mass Spectrometry (DESI MS)-Based Analysis and Imprint Imaging. Anal. Chem..

[23] Tata A., Perez C.J., Hamid T.S., Bayfield M.A., Ifa D.R. (2015). Analysis of Metabolic Changes in Plant Pathosystems by Imprint Imaging DESI-MS. J. Am. Soc. Mass Spectrom..

[24] Wu X., Qin R., Wu H., Yao G., Zhang Y., Li P., Xu Y., Zhang Z., Yin Z., Xu H. (2020). Nanoparticle-immersed paper imprinting mass spectrometry imaging reveals uptake and translocation mechanism of pesticides in plants. Nano Res..

[25] Qin R., Li P., Du M., Ma L., Huang Y., Yin Z., Zhang Y., Chen D., Xu H., Wu X. (2021). Spatiotemporal Visualization of Insecticides and Fungicides within Fruits and Vegetables Using Gold Nanoparticle-Immersed Paper Imprinting Mass Spectrometry Imaging. Nanomaterials.

[26] Chen D., Xu Y., Huang Y., Chen Y., Zhao Y., Yan B., Zhu X., Chen Z., Xu H., Yin Z. (2023). Mapping Molecular Signatures in Plant Leaves, Flowers, and Fruits by a TiO_2_ Nanotube-Based Plasmonic Chip for Imprint Mass Spectrometry Imaging. ACS Agric. Sci. Technol..

[27] Li J., Wei R., Meng Y., Zare R.N. (2024). Evaluation of Oil-Absorbing Film for Imprint Desorption Electrospray Ionization Mass Spectrometry Imaging (IDESI-MSI) on Biological Samples. Metabolites.

[28] Oh B.J., Kim K.D., Kim Y.S. (1999). Effect of Cuticular Wax Layers of Green and Red Pepper Fruits on Infection by *Colletotrichum gloeosporioides*. J. Phytopathol..

[29] Geerdink R.B., Kienhuis P.G.M., Brinkman U.A.T. (1994). Optimization of instrumental parameters for flow injection analysis-thermospray tandem mass spectrometry. Chromatographia.

[30] Race A.M., Bunch J. (2015). Optimisation of colour schemes to accurately display mass spectrometry imaging data based on human colour perception. Anal. Bioanal. Chem..

[31] Wang X., Zheng S., Franceschi P., Liu Y. (2025). Profiling and visualization of organic acids in grape plants by desorption electrospray ionization imaging. Food Chem..

[32] Zhang T., Noll S.E., Peng J.T., Klair A., Tripka A., Stutzman N., Cheng C., Zare R.N., Dickinson A.J. (2023). Chemical imaging reveals diverse functions of tricarboxylic acid metabolites in root growth and development. Nat. Commun..

[33] Baker E.A., Chamel A.R. (1990). Herbicide penetration across isolated and intact leaf cuticles. Pestic. Sci..

[34] Lin R., Xie B., Du C., Hang W., Huang B. (2018). Pulsed micro-discharge ambient ionization mass spectrometry. Int. J. Mass Spectrom..

