# Supplementary material for: Imprint Desorption Electrospray Ionization Mass Spectrometry Imaging (IDESI-MSI) Reveals Absorption of Triclopyr-Based Herbicide in Plants and Mouse Organs
Source: Metabolites. 2025 Jun 30;15(7):437. doi: 10.3390/metabo15070437 (PMC12300929; doi:10.3390/metabo15070437)
Supplement: Supplementary file 1 [file metabolites-15-00437-s001.zip › metabolites-3681649-supplementary.pdf]

Supporting information for

Imprint Desorption Electrospray Ionization Mass Spectrometry Imaging (IDESI-MSI) Reveals Absorption of Triclopyr-based Herbicide in Plants and Mouse Organs

Hanzhi Liu [1,†], Yunshuo Tian [1,†], Ruolun Wei [2], Yifan Meng [3,*] and Richard N. Zare [3,*]

1 College of Letters and Science, University of California, Santa Barbara, CA 93106, USA
2 Department of Neurosurgery, School of Medicine, Stanford University, Stanford, CA 94305, USA; rlwei@stanford.edu
3 Department of Chemistry, Stanford University, Stanford, CA 94305, USA

† These authors contributed equally to this work.

*Correspondence: yfmeng@stanford.edu (Y.M.); zare@stanford.edu (R.N.Z.)

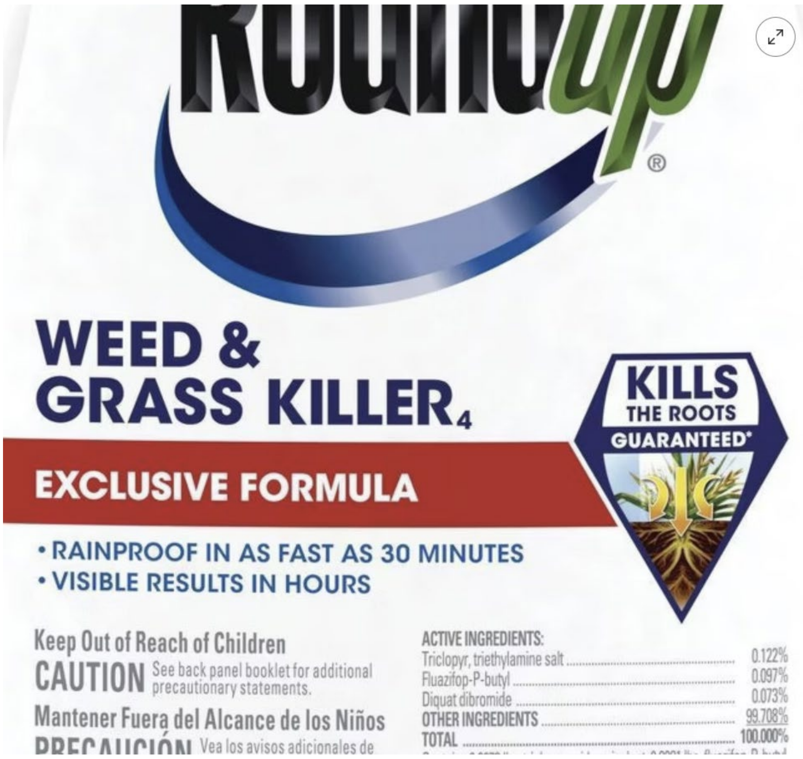

Figure S1. Label of the herbicide that we used in this study.

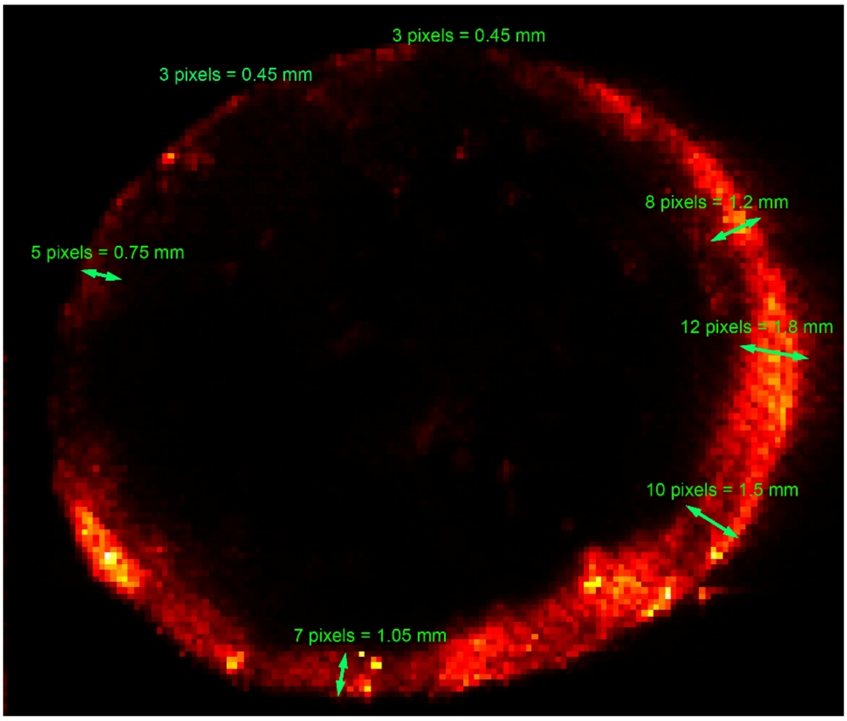

Figure S2. Method for calculating the average penetration depth.

The penetration depth of herbicides is estimated to be based on the spatial distribution coordinates of the herbicide mass spectrometry signals (150 µm/pixel). The maximum penetration was determined by setting a threshold (20%) signal intensity of herbicide and measuring the point at which this signal no longer increases. During the penetration of herbicides into plants, uneven penetration may occur on the same plane. Therefore, the penetration depth of herbicides is simulated by calculating the average depth of multiple penetration paths.

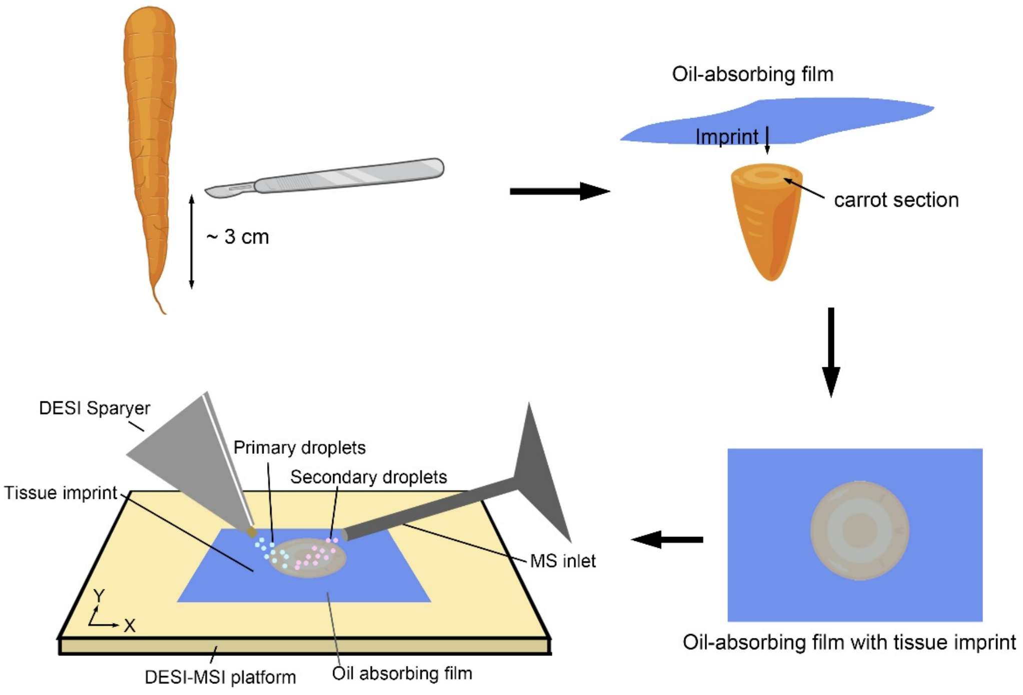

Figure S3. Scheme of the sample preparation and DESI-MSI process.

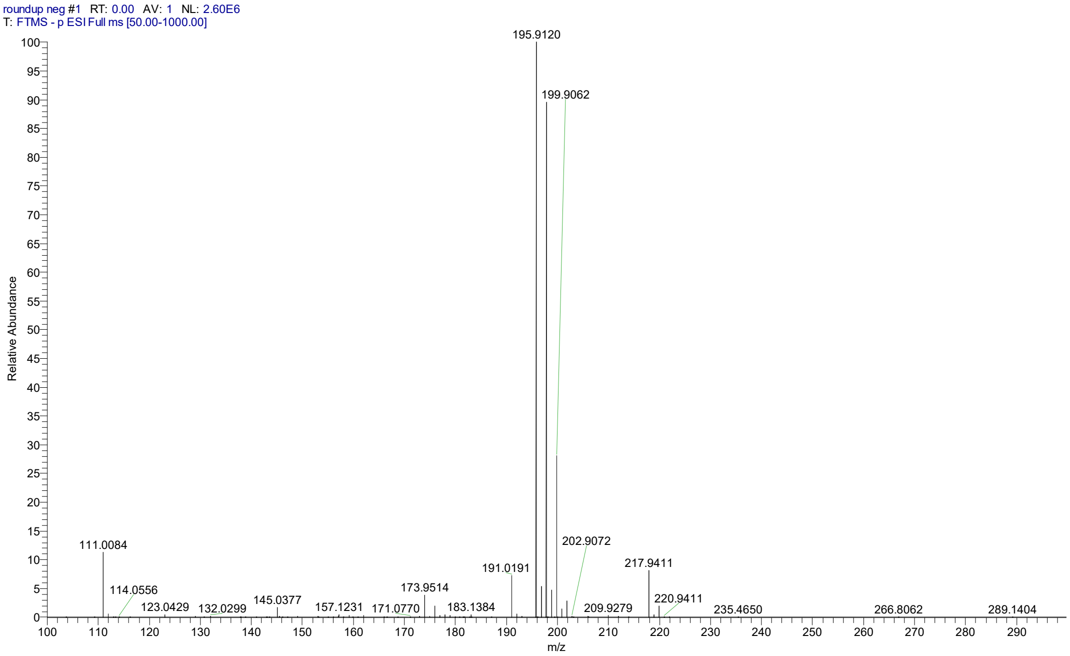

Figure S4. NanoESI mass spectrum of Roundup herbicide in negative ion mode.

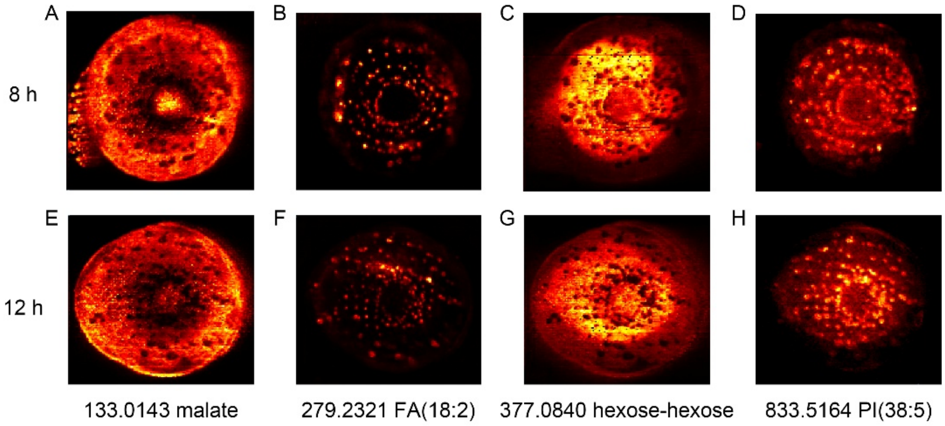

Figure S5. The distribution of malate, FA(18:2), hexose-hexose, and PI(38:5) in carrot exposed to the herbicide for 8 h and 12 h.

Table S1. Theoretical and measured *m/z* of ions detected in this study.

Molecule name	Ion type	Theoretical *m/z*	Measured *m/z*	Relative error (ppm)
Fumarate	$[M-H]^-$	115.0026	115.0035	7.8
Malate	$[M-H]^-$	133.0135	133.0143	6
FA(18:2)	$[M-H]^-$	279.2319	279.2331	4.3
Hexose-hexose	$[M-H]^-$	377.0856	377.0840	-4.2
PI(34:2)	$[M-H]^-$	833.5175	833.5164	-1.3
Triclopyr	$[M-CH_2COOH]^-$	195.9118	195.9124	3.1
Arachidonic acid	$[M-H]^-$	303.2319	303.2330	3.6
PI(18:0/28:4)		885.5487	885.5491	0.3
